# Rspo3 regulates the abnormal differentiation of small intestinal epithelial cells in diabetic state

**DOI:** 10.1186/s13287-021-02385-8

**Published:** 2021-06-07

**Authors:** Ti-Dong Shan, Han Yue, Xue-Guo Sun, Yue-Ping Jiang, Li Chen

**Affiliations:** Department of Gastroenterology, The Affiliated Hospital of Qingdao University, Qingdao University, 16 Jiang Su Road, Qingdao, Shandong 262000 P.R. China

**Keywords:** Diabetes mellitus, Rspos, MicroRNA, Intestinal epithelial stem cell, Differentiation

## Abstract

**Background:**

The complications caused by diabetes mellitus (DM) are the focus of clinical treatment. However, little is known about diabetic enteropathy (DE) and its potential underlying mechanism.

**Methods:**

Intestinal epithelial cells (IECs) and intestinal epithelial stem cells (IESCs) were harvested from BKS.Cg-Dock7^m^+/+Lepr^db^/JNju (DM) mice, and the expression of R-Spondin 3 (Rspo3) was detected by RT-qPCR, Western blotting, immunohistochemistry, and immunofluorescence. The role of Rspo3 in the abnormal differentiation of IECs during DM was confirmed by knockdown experiments. Through miRNA expression profiling, bioinformatics analysis, and RT-qPCR, we further analyzed the differentiation-related miRNAs in the IECs from mice with DM.

**Results:**

Abnormal differentiation of IECs was observed in the mice with DM. The expression of Rspo3 was upregulated in the IECs from the mice with DM. This phenomenon was associated with Rspo3 overexpression. Additionally, Rspo3 is a major determinant of Lgr5+ stem cell identity in the diabetic state. Microarray analysis, bioinformatics analysis, and luciferase reporter assays revealed that microRNA (miR)-380-5p directly targeted Rspo3. Moreover, miR-380-5p upregulation was observed to attenuate the abnormal differentiation of IECs by regulating Rspo3 expression.

**Conclusions:**

Together, our results provide definitive evidence of the essential role of Rspo3 in the differentiation of IECs in DM.

**Supplementary Information:**

The online version contains supplementary material available at 10.1186/s13287-021-02385-8.

## Background

Diabetes mellitus (DM) a very common metabolic disorder, and the complications associated with DM negatively affect the health of millions of people worldwide [1]. Since diabetes-related complications have become the main cause of death worldwide, understanding the pathogenesis of DM has become an important and long-term goal of medical research. Although important progress has been made, there are still many findings that require further study [2]. Previous studies have shown that diabetic enteropathy (DE) is mainly a functional change caused by diabetic autonomic neuropathy, but other precipitating factors, such as inflammation and the microbiota, have recently been identified [3]. However, the abnormal differentiation of intestinal epithelial cells (IECs), as an early change in phenotype, has provided a new understanding of DE. In our previous research, we observed the abnormal proliferation of IECs in diabetic mice [4]. Therefore, the mechanism underlying the abnormal differentiation of IECs was the focus of this study. Previous studies have confirmed that the development of colorectal cancer is highly associated with DM [5, 6], supporting the hypothesis that there may be an association between the abnormal differentiation of IECs and DM.

Intestinal epithelial stem cells (IESCs) undergo dynamic differentiation from the crypts to the villi every 3–10 days. Studies have found that Lgr5+ crypt cells act as stem cells that reside in the crypt base and are essential for the regeneration of normal IECs during homeostasis [7]. The Wnt signaling pathway is a critical component of Lgr5+ crypt cells. In detail, the continuous activation of the Wnt pathway in IESCs causes the excessive activation of β-catenin, which is sufficient to induce IEC polyposis and even cancer [8]. Structurally, R-Spondins 1-4 (Rspos1-4) proteins share a common domain architecture comprising a C-terminal thrombospondin type 1 repeat domain and two furin-like repeats, and the latter is essential for the functions of these proteins in enhancing Wnt signaling [9]. Rspos proteins are secreted amplifiers of Wnt signaling in animals. The sources of these Wnt-amplifying Rspos have been elucidated to understand their functional contribution to intestinal homeostasis [10]. Given the crucial roles of Rspos in the self-renewal of IECs, this study aimed to provide new insight into the functions of Rspos and the underlying mechanism in DE.

MicroRNAs (miRNAs) are conserved noncoding RNAs composed of approximately 18 to 22 nucleotides [11]. miRNAs degrade or inhibit messenger RNA (mRNA) expression by directly binding to the 3′-untranslatable regions (3′-UTRs) of target mRNAs. miRNAs are closely associated with cell proliferation, apoptosis, and differentiation [4, 11]. Abnormal miRNA expression can cause the pathophysiological process of various cancers. However, the role of miRNAs in IECs in DM remains largely unclear and needs to be studied.

In this study, we investigated the role of Rspos in the abnormal IEC differentiation in the diabetic state. To this end, miRNA microarrays and bioinformatic analyses were used to identify candidate miRNAs associated with the abnormal differentiation of IECs in mice with DM to characterize the mechanism by which RSPOs participate in this abnormal differentiation of IECs.

## Methods

### Mice

BKS.Cg-Dock7^m^+/+Lepr^db^/JNju (db/db) mice and BKS heterozygous db/+ mice with an identical genetic background were obtained from the Model Animal Research Center of Nanjing University (Jiangsu, China). Sixteen-week-old mice were individually housed in sterile microisolators for the duration of the experiment. The db/db mice were maintained for 8 weeks with hyperglycemia (random blood glucose levels ≥ 16.7 mmol/l). In addition, db/+ mice (random blood glucose levels < 11.1 mmol/l) were used as the controls [12]. All the experimental procedures were approved by the Animal Care Committee of Qingdao University.

### Reverse transcription-quantitative PCR (RT-qPCR) analysis

Total RNA was extracted from cell lines and tissue samples with TRIzol Reagent (Invitrogen; Thermo Fisher Scientific, Inc., CA, USA) following the manufacturer’s manual. Then, RT-qPCR was performed with PrimeScript™ RT Master mix and qPCR SYBR® Premix Ex Taq™ (Takara Biotechnology Co., Ltd., Dalian, China). The following thermocycling conditions were used for the qPCRs: denaturation at 95 °C for 7 min, followed by 40 cycles of denaturation at 95 °C for 15 s and 60 °C for 1 min. The miRNA levels were detected by a SYBR PrimeScript™ miRNA RT-PCR kit (Takara Biotechnology Co., Ltd., Dalian China). The primers that were used are listed in Table S1. The RNA levels were calculated using the 2-CqΔΔ method [13].

### Bioinformatics analysis

miRNAs that potentially bind to the 3′-UTR of Rspo mRNA were predicted using 2 different algorithms between TargetScan 7.2 (http://www.targetscan.org/) and miRanda (http://www.miranda.org).

### Culture of cell lines

293T cells were obtained from American Type Culture Collection and were cultured in DMEM (Invitrogen; Thermo Fisher Scientific, Inc., CA, USA) supplemented with 10% FBS (Gibco; Thermo Fisher Scientific, Inc., CA, USA) and 1% penicillin/streptomycin and streptomycin (0.1 mg/ml; Sigma-Aldrich; Merck KGaA) at 37 °C with 5% CO_2_.

### Primary IEC isolation

The small intestines were harvested from the mice, and the crypts and villi were exposed through longitudinal slices. All the procedures were performed as described previously [12, 14–16].

### Cell transfection

siRNAs targeting Rspo3, a miRNA mimic (agomiR-380-5p), and an inhibitor (antagomiR-380-5p) were synthesized and purchased from Guangzhou RiboBio Co., Ltd. (China). Cells (2 × 10^5^ cells/well) were seeded into six-well plates and incubated overnight prior to transfection. After the reached 40–60% confluence, the cells were transfected with siRNAs (15 nM), a miRNA mimic (15 nM), or an inhibitor (15 nM) using Lipofectamine 3000 (Invitrogen; Thermo Fisher Scientific, Inc., CA, USA).

### Dual-luciferase reporter plasmid transfection

The wild-type (WT) or mutant (MUT) 3′-UTR of the Rspo3 sequence was cloned into the pmiR-RB-REPORT™ plasmid (Guangzhou RiboBio Co., Ltd., Guangzhou, China). After incubation for 48 h, the cells were collected, and the firefly and Renilla luciferase activities were measured using the Dual-Luciferase Reporter Assay System (Promega Corporation, Madison, WI, USA). The firefly luciferase activity was normalized to the Renilla luciferase activity. The luciferase efficiency was evaluated 2 min after the addition of the Stop & Glo® reagent using a SpectraMAX Multifunctional Microplate Reader (Molecular Devices, USA).

### Downregulating the expression of Rspo3 in vivo

The mice were randomly divided into four groups, with 24 mice in each group. All the mice received a tail vein injection once a day for 3 days. The db/+-NS group included control mice that received saline injections (0.9%; same volume as the experimental group) [14, 15, 17, 18]; the db/db-NS group included mice with DM that received saline injections (0.9%; same volume as the experimental group) [14, 15, 17, 18]; the db/db-si*-*Rspo3 group included mice that received injections of Rspo3 stable TM siRNA (stable TM siRNA is primarily used in vivo as a long-acting, chemically modified siRNA, Guangzhou RiboBio Co., Ltd.); 80 mg/kg body weight, [14, 15, 17, 18], and the db/db-agomiR-380-5p group included mice that received injections of agmiR-380-5p (80 mg/kg body weight, [14, 15, 17, 18]). In each group, six mice were euthanized with an intraperitoneal injection of ketamine/xylazine (100/10 mg/kg body weight) on day 0 (prior to injection), day 2, day 4, and day 6, and tissues were harvested for further study.

### miRNA microarray and data analysis

Analysis of miRNA expression profiling was obtained from Kangcheng Biological Company (Shanghai). Briefly, total miRNAs were extracted from IECs using an miRNA isolation kit (Takara, Otsu, Shiga, Japan), and according to the manufacturers’ guidelines for the miRCURYTM Hy3TM/Hy5TM Power labeling kit (Exiqon, Vedbaek, Denmark), the samples were labeled and hybridized on a miRCURYTM LNA Array (v.18.0; Exiqon). After the washing steps, the slides were scanned using the Axon GenePix 4000B microarray scanner. Scanned images were then imported into GenePix Pro 6.0 software (Axon) for grid alignment and data extraction. The data from replicate miRNAs were averaged, and miRNAs with intensities ≥ 30 in all the samples were chosen to calculate the normalization factor. The expressed data were normalized using median normalization. After normalization, significant differentially expressed miRNAs were identified by volcano plot filtering. Finally, hierarchical clustering was performed to reveal distinguishable miRNA expression profiles among the samples.

### In situ hybridization

A DIG-labeled LNA-miR-380-5p probe was synthesized by RiboBio following the manufacturer’s instructions (Guangzhou, China). In brief, a 5-mm section of paraffin-embedded tissue was incubated with methanol in PBST, fixed with 4% formaldehyde solution, washed with SSC buffer, and then permeabilized with Triton X-100 solution. The tissues were incubated with the DIG-labeled LNA-miR-380-5p probe for hybridization at 37 °C overnight. Then, miR-380-5p expression was determined using diaminobenzidine solution (1:900; Boster Biological Technology, Wuhan, China), and the staining intensity was observed using a BX51 microscope (Olympus Corporation, Tokoyo, Japan). The staining was quantified by counting the number of positive cells at a magnification of × 400.

### Immunohistochemistry

The paraffin-embedded mouse tissue samples were sectioned at a thickness of 5 μm. The sections were deparaffinized in xylene and rehydrated in an ethanol gradient. The tissue sections were blocked with a peroxidase-blocking solution (Dako, Glostrup, Denmark). Then, the samples were incubated in milk for 5 min and overnight at 4 °C with primary antibodies, including anti-SI antibody (1:100; cat no. ab84977), anti-Tff3 antibody (1:150; cat no. sc398651), anti-Lyz1 antibody (1:150; cat no. ab189937), anti-ChgA antibody (1:100; cat no. ab254322), anti-Rspo1 antibody (1:200; cat no. ab106556), anti-Rspo2 antibody (1:100; cat no. ab132836), anti-Rspo3 antibody (1:150; cat no. ab233113), and anti-Rspo4 antibody (1:100; cat no. ab189515) (all from Abcam, Inc., Cambridge, MA, USA). For immunohistochemistry, the sections were incubated with anti-HRP rabbit/mouse secondary antibodies (Dako, Glostrup, Denmark) at room temperature for 2 h, and the color was visualized with DAB (Dako, Glostrup, Denmark). The sections were stained with Mayer’s hematoxylin solution, dehydrated with xylene, and observed under a microscope (Olympus, Tokyo, Japan).

### Protein extraction and Western blotting

Total protein was extracted from the tissues using RIPA buffer (Thermo Fisher Scientific, Inc., CA, USA) supplemented with a protease inhibitor cocktail (Roche Applied Science, CA, USA). The protein samples (40 μg/sample) from each group were loaded and resolved on 10% SDS-PAGE gels and subsequently transferred to the polyvinylidene fluoride (PVDF) membranes (EMD Millipore, Temecula, CA, USA). Then, the membranes were blocked with 5% skim milk at room temperature for 1 h and incubated at 4 °C overnight with the following primary antibodies: anti-SI antibody (1:400; cat no. ab84977), anti-Tff3 antibody (1:400; C cat no. sc398651), anti-Lyz1 antibody (1:250; cat no. ab189937), anti-ChgA antibody (1:200; cat no. ab254322), anti-Rspo1 antibody (1:1000; cat no. ab106556), anti-Rspo2 antibody (1:1000; cat no. ab132836), anti-Rspo3 antibody (1:1000; cat no. ab233113), anti-Rspo4 antibody (1:1000; cat no. ab189515), and anti-β-actin antibody (1:1000; cat no. ab8226) (all from Abcam, Inc., Cambridge, MA, USA). The blots were incubated with horseradish peroxidase-conjugated secondary antibodies (cat no. 7074S; Cell Signaling Technology, Inc., Danvers, MA, USA) at 37 °C for 1 h at room temperature and visualized using an enhanced chemiluminescence Ultra Western HRP Substrate kit (cat no. WBULS0100; EMD Millipore, Temecula, CA, USA). The signals were analyzed by the Quantity One software version 4.6.2 (Bio-Rad Laboratories, Inc., Minneapolis, MN, USA), and the intensity values were normalized to β-actin.

### Statistical analysis

The data are presented as the mean ± standard deviation and were analyzed using the Statistical Software Package SAS 8.0 for Windows (SAS Institute, Inc., Cary, NC, USA). Comparisons between the groups were analyzed with Student’s test, and one-way ANOVA with Tukey’s post hoc test was used for multiple group comparisons. *P* < 0.05 was considered the criterion for statistical significance.

## Results

### Abnormal differentiation of IECs in DM state

**Fig. 1:**
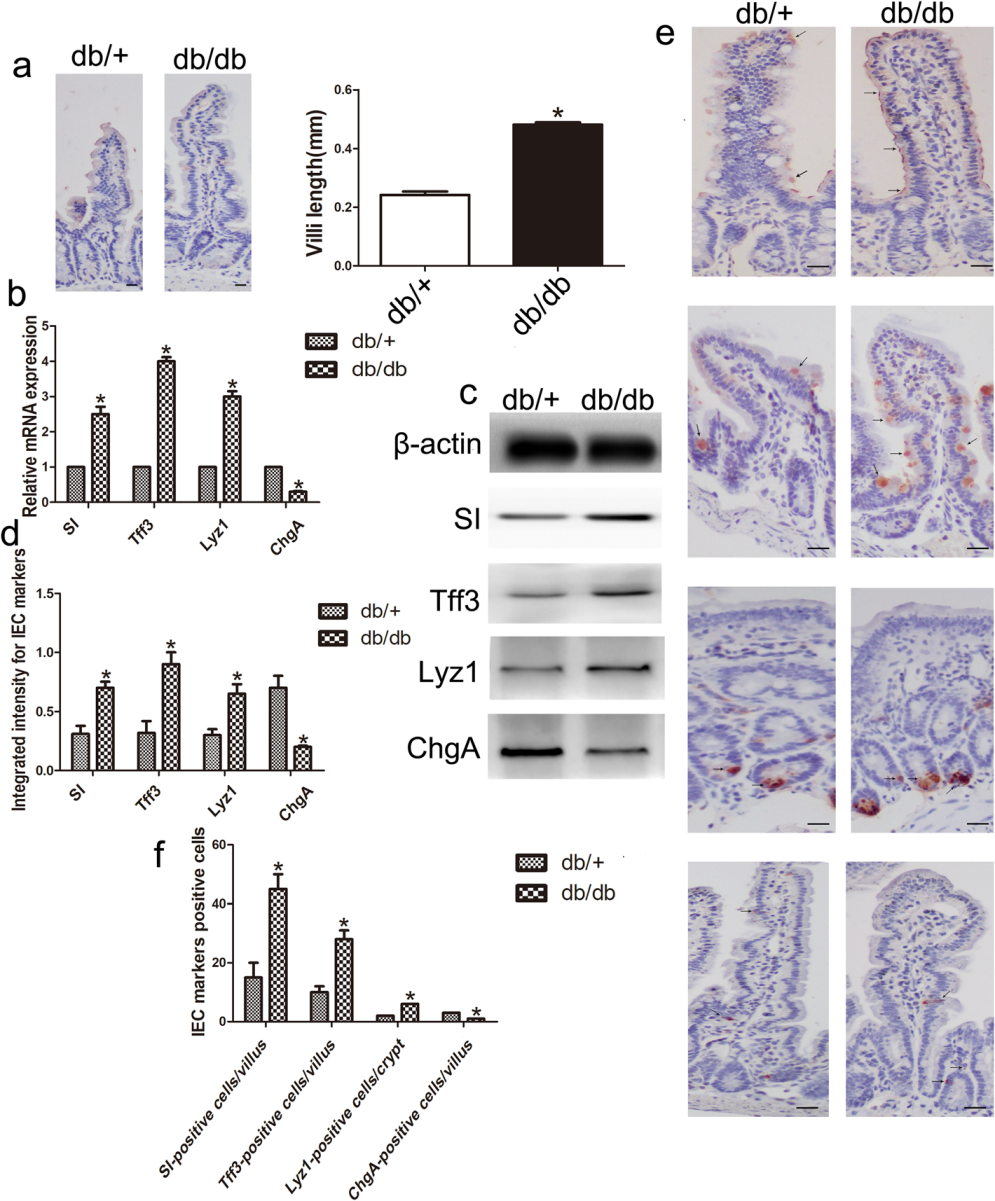
Abnormal differentiation of IECs in DM. **a** Hematoxylin staining was used to detect the length of the villi. **b** The mRNA expression levels of SI, Tff3, Lyz1, and ChgA in the IECs of the db/db group and the db/+ group. **c**, **d** Western blot analysis showed the protein expression of SI, Tff3, Lyz1, and ChgA in the two groups. **e**, **f** Immunohistochemistry showed the numbers of SI-, Tff3-, Lyz1-, and ChgA-positive cells. Scale bar, 50 μm; mean ± SD, n = 6; *P < 0.05

Hematoxylin staining was used to detect the length of the villi. The results showed that the villus length was significantly increased in the db/db mice compared with the control mice (n = 6, *P* < 0.05; Fig. 1a). Sucrase-isomaltase (SI), trefoil factor 3 (Tff3), lysozyme 1 (Lyz1), and chromogranin A (ChgA) were used as markers of absorptive cells, goblet cells, Paneth cells, and endocrine cells, respectively. In this study, RT-qPCR analysis revealed that the IECs from the db/db mice had an abnormal differentiation profile compared with those from the db/+ mice. The results showed the overexpression of SI, Tff3, and Lyz1 in the db/db mice compared with the db/+ mice (n = 6, *p* < 0.05; Fig. 1b); however, there was lower expression of ChgA (n = 6, *p* < 0.05; Fig. 1b). Western blot analysis was also used to investigate protein expression levels (Fig. 1c). The protein expression of SI, Tff3, and Lyz1 was significantly increased, and the protein expression of ChgA was decreased, in the db/db mice (n = 6, *p* < 0.05; Fig. 1d). Furthermore, immunohistochemistry showed that the numbers of SI-, Tff3-, and Lyz1-positive cells in the db/db mice were significantly increased, and the numbers of ChgA-positive cells were decreased compared with those in the db/+ mice (n = 6, *p* < 0.05; Fig. 1e, f). Taken together, these results suggest the abnormal differentiation of IECs in DM.

### Rspo3 is overexpressed in the IECs of mice with DM

**Fig. 2:**
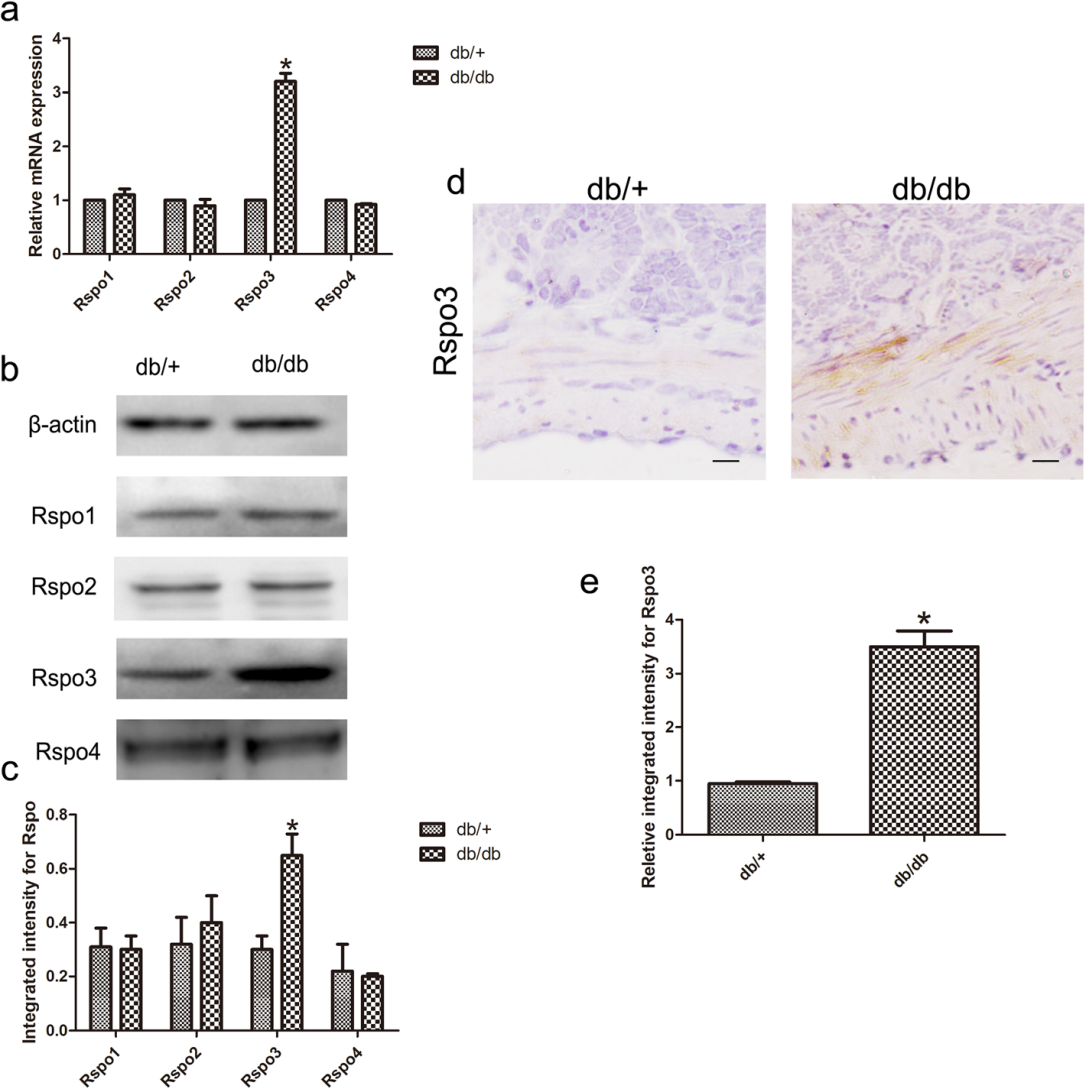
The expression of Rspo3 in the IECs in DM. **a** RT-qPCR analysis showed the mRNA expression of Rspos in the IECs of the db/db mice and the db/+ mice. **b**, **c** Rspo3 protein levels were also significantly upregulated in the IECs of the db/db mice. **d**, **e** Immunohistochemistry showed that Rspo3 expression was localized around the crypt base. Scale bar, 50 μm; mean ± SD, n = 6; *P < 0.05

To study the role of four Rspo family members in IECs of mice with DM, the expression profiles of these proteins were detected in db/db mice. Subsequently, RT-qPCR analysis showed that of these proteins, Rspo3 showed higher expression in the IECs of the db/db mice than in those of the db/+ mice (n = 6, *p* < 0.05; Fig. 2a). The Rspo3 protein levels were also significantly upregulated in the IECs of the db/db mice (n = 6, *p* < 0.05; Fig. 2b, c). As shown by immunohistochemistry, we found that Rspo3 expression was predominantly localized in the stromal compartment that surrounds the crypt base. The expression of Rspo3 in the db/db mice was significantly increased (n = 6, *p* < 0.05; Fig. 2d, e). Overall, we hypothesized that Rspo3 plays an important role in the abnormal differentiation of IECs in mice with DM.

### Abnormal differentiation of IECs in DM is associated with Rspo3 overexpression

**Fig. 3:**
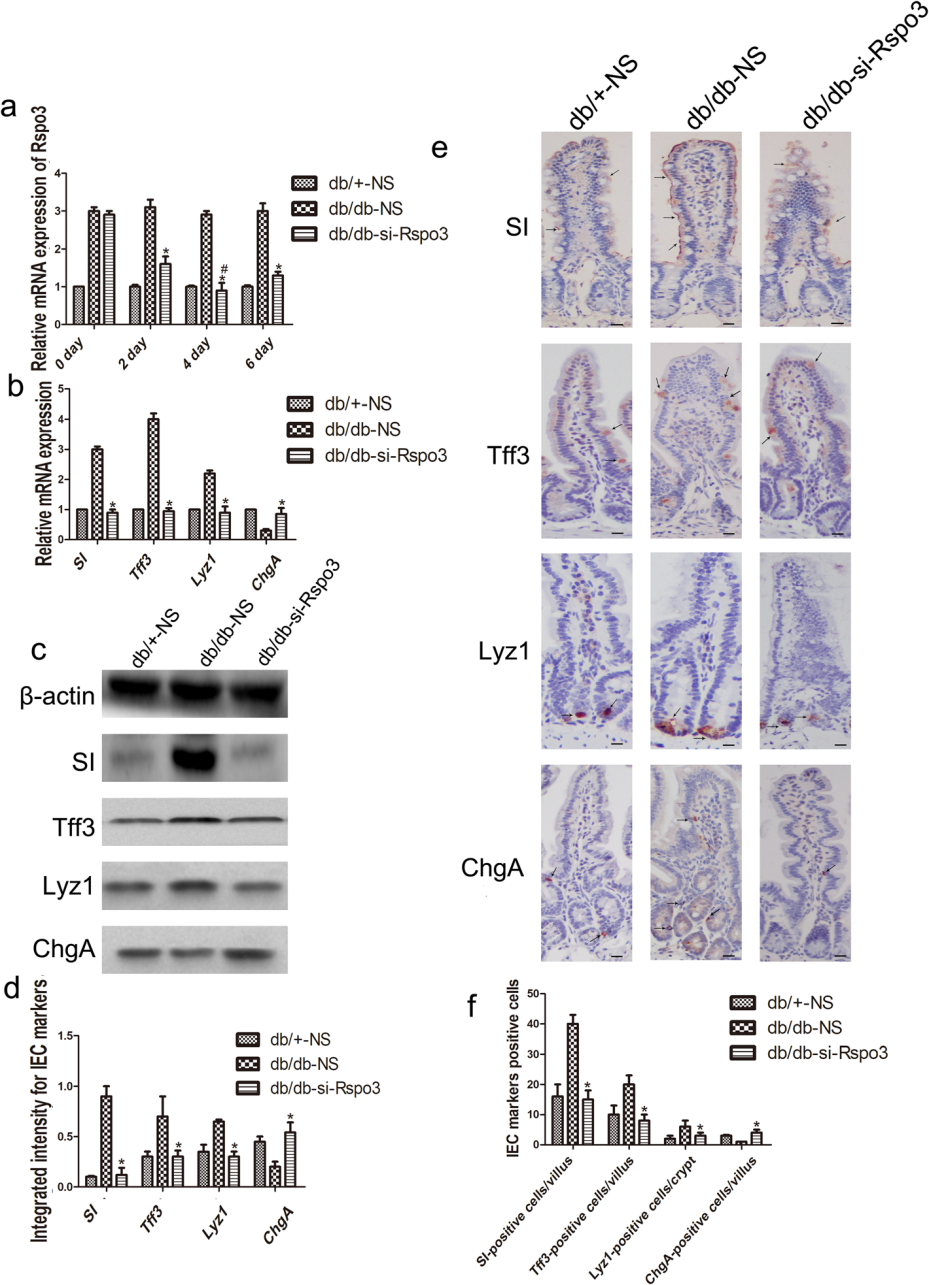
Abnormal differentiation of IECs in the DM state is associated with overexpression of Rspo3. **a** The knockdown efficiencies after transfection of siRNAs in vitro. **b** The mRNA expression of SI, Tff3, Lyz1, and ChgA after si-Rspo3 transfection into the IECs of the db/db mice. **c**, **d** Western blot analysis showed the protein expression of SI, Tff3, Lyz1, and ChgA in the si-Rspo3-treated db/db mice. **e**, **f** The numbers of SI-, Tff3-, Lyz1-, and ChgA-positive cells in the si-Rspo3-administered db/db mice. Scale bar, 50 μm; mean ± SD, n = 6; *P < 0.05 vs db/+-NS

Rspo3 expression in the IECs of the stable TM si-Rspo3-treated db/db mice was significantly reduced at 2, 4, and 6 days compared to that in the IECs of the db/db mice (n = 6, *p* < 0.05; Fig. 3a), and the expression of Rspo3 on the 4th day after administration was similar to that in the IECs from the db/+ mice (n = 6, *p* > 0.05; Fig. 3a). On the 4th day after stableTM si-Rspo3 administration, the increased mRNA expression of SI, Tff3, and Lyz1 was inhibited in the db/db mice, and the mRNA expression of ChgA was increased (n = 6, *p* < 0.05; Fig. 3b). This phenotype was further confirmed by Western blot, and the protein expression of SI, Tff3, and Lyz1 in the si-Rspo3-treated db/db mice was significantly decreased and close to the levels observed in the db/+ mice (n = 6, *p* < 0.05; Fig. 3c, d). Furthermore, the downregulated ChgA protein expression in the db/db mice was partially normalized after si-Rspo3 administration and comparable to the expression in the db/+ mice (n = 6, *p* < 0.05; Fig. 3c, d). Moreover, after treatment of the db/db mice with si-Rspo3, the numbers of SI-, Tff3-, and Lyz1-positive cells were significantly decreased, and the decreased numbers of ChgA-positive cells normalized (n = 6, *p* < 0.05; Fig. 3e, f). These results suggest that Rspo3 might be capable of promoting the abnormal differentiation of IECs in mice with DM.

### Knockdown of RSPO3 reduced the overexpressed Lgr5+ stem cell identity in the DM state

**Fig. 4:**
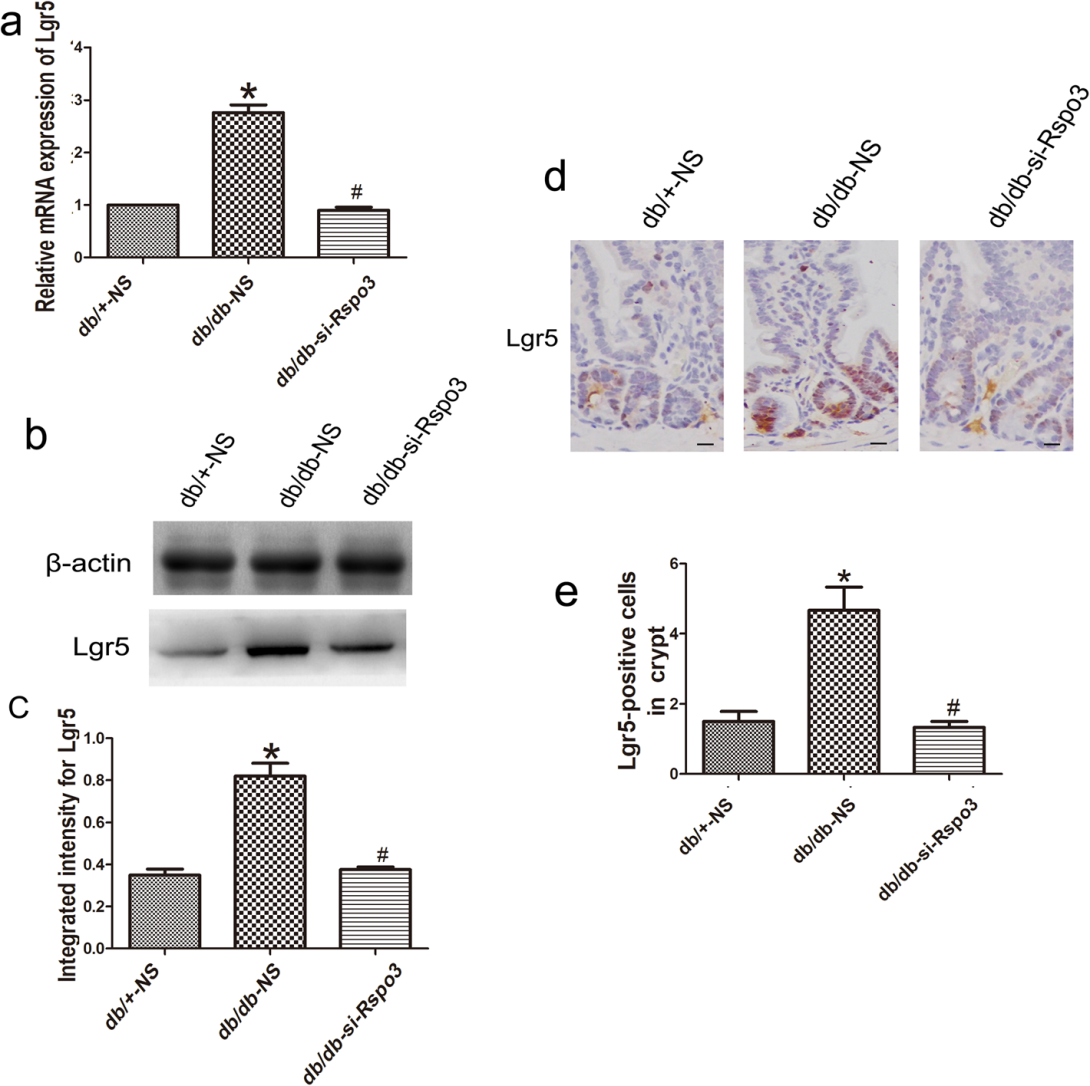
RSPO3 expands Lgr5+ stem cells in the DM state. **a** RT-qPCR revealed that Lgr5 in the db/db-NS mice showed higher expression than that in the db/+-NS mice, and the high Lgr5 expression was decreased after si-Rspo3 administration. **b**, **c** Western blot analysis showed that the Lgr5 protein levels were upregulated and downregulated in the si-Rspo3-treated db/db mice. **d**, **e** Immunohistochemistry showed that Lgr5 expression was predominantly localized in the crypts of IECs and that Lgr5+ stem cell zones were expanded in the db/db-NS mice. After si-Rspo3 administration in the db/db-NS mice, high Lgr5 expression was inhibited, and the expansion of the Lgr5+ stem cell zone was reduced. Scale bar, 50 μm; mean ± SD, n = 6; *P < 0.05 vs db/+-NS; ^#^*P* < 0.05 vs db/db-NS

As shown in Fig. 4a, RT-qPCR analysis revealed that Lgr5 expression was higher in the db/db-NS mice than in the db/+-NS mice (n = 6, *p* < 0.05; Fig. 4a). The Lgr5 protein levels were also significantly upregulated, as shown by Western blot analysis (n = 6, *p* < 0.05; Fig. 4b, c). Immunohistochemistry showed that Lgr5 expression was predominantly localized in the crypts of the IECs and that the Lgr5+ stem cell zone was expanded in the db/db-NS mice (n = 6, *p* < 0.05; Fig. 4d, e). After treatment of the db/db-NS mice with si-Rspo3, the high Lgr5 expression was inhibited, and the expansion of the Lgr5+ stem cell zone was reduced, as shown by RT-qPCR, Western blot, and immunohistochemical analyses (n = 6, *p* < 0.05; Fig. 4a–e). Together, these data indicate that Rspo3 is a major determinant of Lgr5+ stem cell identity in the DM state.

### miRNA expression profiles were evaluated in the IECs of mice with DM

**Fig. 5:**
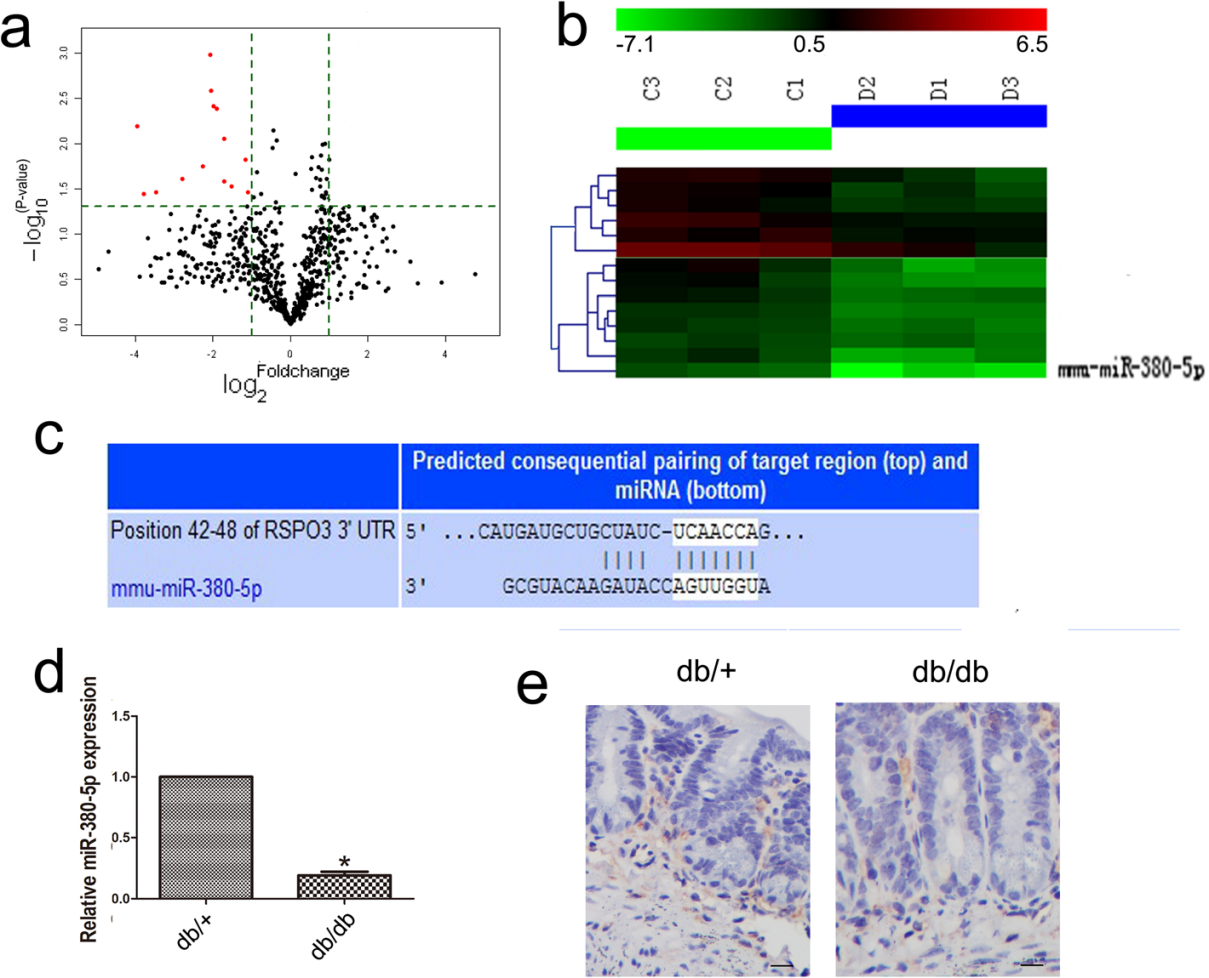
miRNA expression profiles were evaluated in the IECs from mice with DM. **a** The red point in the plot represents the differentially expressed miRNAs with statistical significance in the volcano plot. **b** Microarray analysis was used to evaluate the miRNA expression profiles in the db/db mice. C, control mice; D, diabetic mice. **c** Rspo3 targets Rspo3, as shown by an analysis of publicly available algorithms. **d** RT-qPCR revealed that miR-380-5p expression was downregulated in the IECs of the db/db mice. **e** In situ hybridization with a DIG-labeled LNA-miR-380-5p probe showed that miR-380-5p was predominantly localized near the crypt base. Scale bar, 50 μm; mean ± SD, n = 6; *P < 0.05

To gain a broader understanding of how Rspo3 is upregulated in IECs in DM, we performed microarray analysis. Microarray analysis was used to evaluate miRNA expression profiles; volcano plot and hierarchical clustering identified 14 miRNAs that were downregulated in the IECs of the db/db mice compared to those of the db/+ mice (Fig. 5a, b and Table S1). Among these miRNAs, miRNA-380-5p, which may target Rspo3, was considered to be a candidate for further investigation by analyzing publicly available algorithms (TargetScan, www. targetscan.org; miRanda, www.microrna.org; Fig. 5c). RT-qPCR further revealed that miR-380-5p expression in the IECs of the db/db mice was significantly downregulated compared to that in the IECs of the db/+ mice (n = 6, P < 0.05; Fig. 5d). Moreover, in situ hybridization with a DIG-labeled LNA-miR-380-5p probe showed that miR-380-5p was predominantly localized in the stromal compartment that surrounds the crypt base (n = 6, P < 0.05; Fig. 5e).

### MiR-380-5p regulates the abnormal differentiation of IECs via Rspo3 in DM

**Fig. 6:**
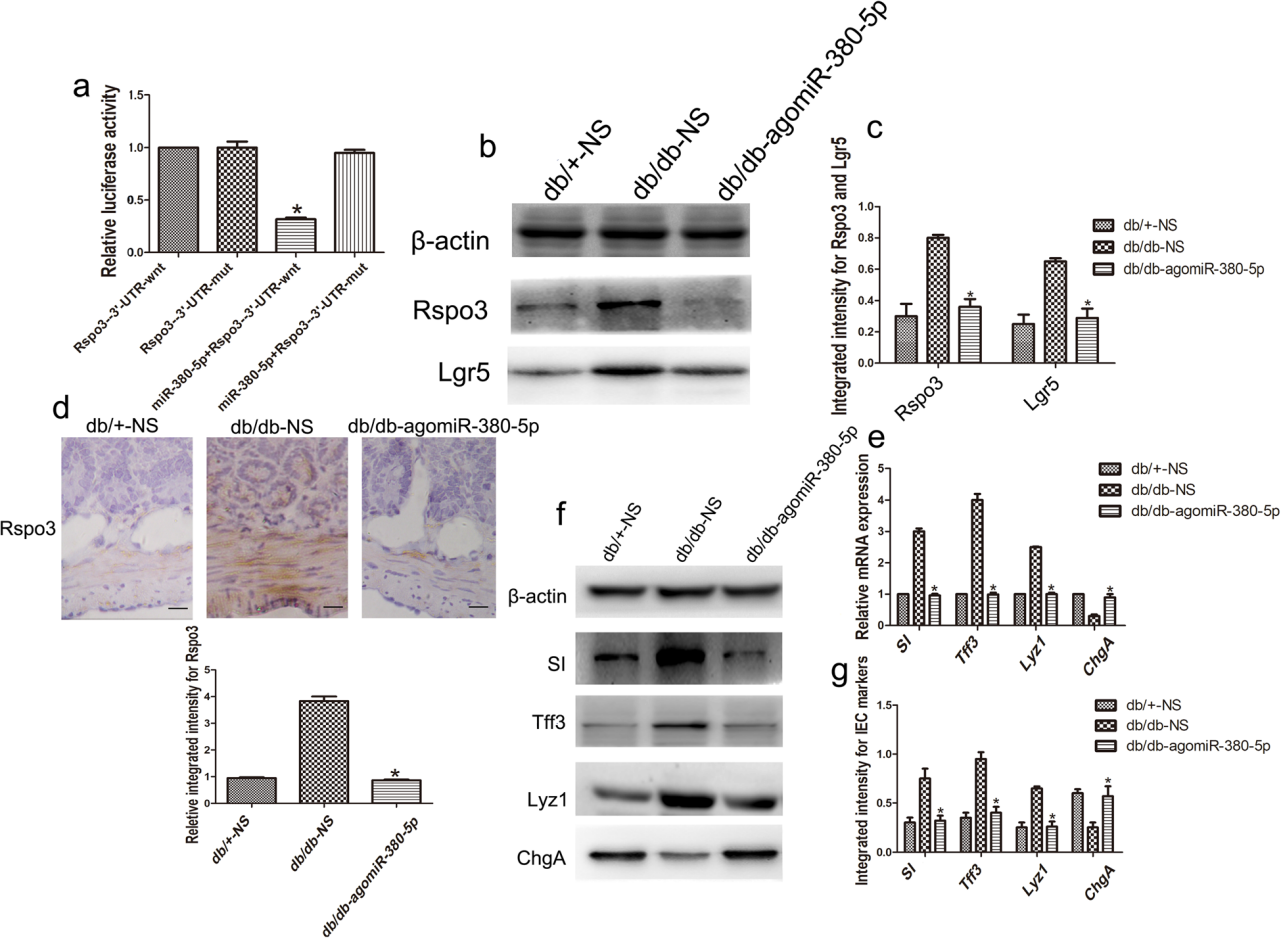
MiR-380-5p regulates the abnormal differentiation of IECs via Rspo3 in the DM state. **a** The dual-luciferase reporter assay demonstrated that the miR-380-5p mimic and the Rspo3-3′-UTR-wt plasmid caused a significant decrease in the luciferase activity. **b**, **c** AgomiR-380-5p administration in the db/db mice reduced the overexpressed levels of Rspo3 and Lgr5 after agomiR-380-5p administration in the db/db mice, as shown by Western blot. **d** Immunohistochemistry showing Rspo3 expression in the db/db mice after agomiR-380-5p administration. **e** RT-qPCR showed substantially increased mRNA expression of SI, Tff3, and Lyz1 and decreased mRNA expression of ChgA in the db/db mice, which was rescued by the administration of agomiR-380-5p. **f**, **g** SI, Tff3, and Lyz1 protein overexpression was substantially decreased, and the levels were similar to the levels observed in the db/+ mice after agomiR-380-5p administration. Additionally, the downregulated ChgA protein expression was partially normalized after agomiR-380-5p administration to levels that were comparable to those observed in the db/+ mice. Scale bar, 50 μm; mean ± SD, n = 6; *P < 0.05 vs db/+-NS; ^#^*P* < 0.05 vs db/db-NS

The dual-luciferase reporter assay demonstrated that cotransfection of 293T cells with the miR-380-5p mimic and the Rspo3-3′-UTR-wnt plasmid resulted in a significant decrease in the luciferase activity (n = 6, *p* < 0.05; Fig. 6a). The luciferase activity of the Rspo3-3′-UTR-mut plasmid was not affected by the miR-380-5p mimic (n = 6, *p* > 0.05; Fig. 6a). These data indicate that the miR-380-5p mimic specifically targeted the 3′-UTR of Rspo3 and decreased the expression of the downstream reporter gene. Treatment of db/db mice with agomiR-380-5p reduced the overexpression levels of Rspo3 and Lgr5, as shown by Western blot (n = 6, *p* < 0.05; Fig. 6b, c). As shown by immunohistochemistry, we found that the Rspo3 expression that was localized to the stromal compartment in db/db mice was significantly decreased after agomiR-380-5p administration (n = 6, *p* < 0.05; Fig. 6d). Interestingly, RT-qPCR showed substantially increased mRNA expression of SI, Tff3, and Lyz1 and decreased mRNA expression of ChgA in the db/db mice, which was rescued by the administration of agomiR-380-5p (n = 6, *p* < 0.05; Fig. 6e). Furthermore, the SI, Tff3, and Lyz1 protein overexpression was decreased by agomiR-380-5p administration, and these levels were close to the levels observed in the db/+ mice (n = 6, *p* < 0.05; Fig. 6f, g). Furthermore, the downregulated ChgA protein expression was partially normalized after agomiR-380-5p administration, and the levels were comparable to those observed in the db/+ mice (n = 6, *p* < 0.05; Fig. 6f, g).

## Discussion

DM-associated complications that affect the gastrointestinal tract have already received increasing attention. We primarily focused on the clinical treatment of these complications with an emphasis on the major symptoms, including diarrhea, constipation, and anal sphincter incontinence. In fact, before the symptoms mentioned above appear, the intestinal epithelium has already undergone abnormal proliferation and differentiation. The aim of this study was to evaluate the abnormal differentiation of IECs in db/db mice. In this study, we found that the numbers of SI-, Tff3-, and Lyz1-positive cells in db/db mice were significantly increased, and the numbers of ChgA-positive cells were decreased. This was consistent with the abnormal differentiation observed in the intestinal epithelium of a streptozocin (STZ)-induced DM mouse model in our previous study [15]. Type 1 diabetes and type 2 diabetes have different etiologies, pathogeneses, and clinical manifestations. Mice with STZ-induced DM are often considered a type 1 diabetes model; on the other hand, db/db mice are often considered a type 2 diabetes model. In this study, it was found that these mice had the same abnormal differentiation of the intestinal epithelium at the beginning of DE, but whether it was always the same needs to be further studied.

To continue our research, the elucidation of the molecular mechanisms that regulate the differentiation of IECs and their function would be crucial for understanding the pathogenesis of intestinal disease in the DM state. IESCs rapidly proliferate and differentiate into mature epithelial cells to maintain intestinal integrity. Lgr5+ crypt cells, such as IESCs, that reside in crypts are critical for continuous self-renewal and are thought to be indispensable for small intestinal (SI) homeostasis [19]. In our study, we found that Lgr5 was upregulated and that the Lgr5+ stem cell zone was expanded in db/db mice compared with those in db/+ mice, and these findings explain the main cause of the abnormal differentiation of IECs in the DM state.

In our previous research, we found that the Wnt/β-catenin signaling pathway was continuously activated in the IECs of mice with DM [14], and the reason why the Wnt/β-catenin pathway was continuously activated still needs further study. In IESCs, the Wnt pathway is essential for intestinal crypt formation and renewal, whereas Rspo-mediated signaling mainly affects ISC numbers [20]. All Rspos have the capacity to induce crypt cell renewal and β-catenin activation [21]. In most previous studies, the Rspo3-knockout model revealed that Rspo3 is an indispensable factor for the regeneration of intestinal epithelial stem cells [22, 23]. However, a study of an Rspo3 overexpression model showed that Rspo3 overexpression can cause the occurrence and development of colon cancer by excessively activating the WNT pathway [24]. In this study, we first observed that among the Rspo family members (Rspo1-4), Rspo3 was significantly upregulated in IECs in the DM state. We found that Rspo3 is a major determinant of Lgr5+ stem cell identity in the DM state. Furthermore, the results showed that the numbers of SI-, Tff3-, and Lyz1-positive cells were significantly decreased, and the decreased numbers of ChgA-positive cells observed in db/db mice were normalized after si-Rspo3 administration. These data suggest that the abnormal differentiation of IECs in the DM state is associated with the overexpression of Rspo3.

Given its crucial role in the differentiation of IECs, investigating the mechanism underlying the action of Rspo3 has provided new insight into its regulatory roles in stem cell behavior. Many studies have demonstrated that miRNAs play roles in the differentiation of IECs in mice [14, 25, 26]. Through miRNA expression profiling, bioinformatics analysis, and RT-qPCR, we identified an miRNA, miR-380-5p, that was downregulated in IEC tissues. In addition, by in situ hybridization with a DIG-labeled LNA-miR-380-5p probe, we found that the distribution of miR-380-5p was consistent with that of Rspo3. MiR-380-5p has been found to be able to regulate the development of different tumors [27, 28]. However, under diabetic conditions, whether miR-380-5p also plays a role in the differentiation of IECs needs to be elucidated. In this study, luciferase analysis showed that miR-380-5p targeted the Rspo3 gene and downregulated the expression of the downstream reporter gene. Given the common target identified in this work, it would be interesting for further studies to determine the extent to which miR-380-5p functions in the abnormal differentiation of IECs.

Numerous studies have shown that miRNAs can improve intestinal function by participating in the differentiation of cell populations in the intestine [29]. The miR-30 family controls the differentiation of IECs by regulating the expression of a wide range of genes that includes SOX9 and genes associated with the ubiquitin ligase pathway [30]. However, there are few studies about whether miRNAs regulate the abnormal differentiation of the intestinal epithelium in the DM state, and this study aimed to address this question. In this study, the numbers of SI-, Tff3- and Lyz1-positive cells were significantly decreased, and the decreased number of ChgA-positive cells observed in db/db mice was normalized after agomiR-380-5p administration. These results suggest that miR-380-5p regulates the abnormal differentiation of IECs via Rspo3 in the DM state.

## Conclusions

In conclusion, this study provides new evidence that Rspo3 plays a significant role in regulating the differentiation of IECs in DM. Further analyses showed that Rspo3 is a major determinant of Lgr5+ stem cell identity in the DM state. Decreased expression of miR-380-5p appears to be a key player in this pathological process by targeting Rspo3.

## Supplementary Information

**Additional file 1: Table S1.** List of differentially expressed miRNAs in IECs of DM mice compared with normal control mice.

## Data Availability

All the data generated or analyzed during this study are included in this published article.

## Abbreviations

DM: Diabetes mellitus
DE: Diabetic enteropathy
Rspo: R-spondin
miRNA: MicroRNA
mRNA: Messenger RNA
IECs: Intestinal epithelial cells
IESCs: Intestinal epithelial stem cells
qPCR: Quantitative polymerase chain reaction
UTR: Untranslated region
