# Supplementary material for: Rspo3 regulates the abnormal differentiation of small intestinal epithelial cells in diabetic state
Source: Stem Cell Res Ther. 2021 Jun 7;12:330. doi: 10.1186/s13287-021-02385-8 (PMC8186182; doi:10.1186/s13287-021-02385-8)
Supplement: Supplementary file 1 — Additional file 1: Table S1. List of differentially expressed miRNAs in IECs of DM mice compared with normal control mice. [file 13287_2021_2385_MOESM1_ESM.docx]

ESM Table 1: List of differentially expressed miRNAs in IECs of DM mice compared with normal control mice.

*Down-regulated miRNAs*

| miRNA | Fold change (Ratio ≥ 2) | P-value |
| --- | --- | --- |
| mmu-miR-719 | 0.238716 | 0.001052 |
| mmu-miR-668-3p | 0.268827 | 0.004156 |
| mmu-miR-434-3p | 0.090418 | 0.034908 |
| mmu-miR-380-5p | 0.064812 | 0.006462 |
| mmu-miR-2136 | 0.46712 | 0.03466 |
| mmu-miR-434-5p | 0.072963 | 0.036102 |
| mmu-miR-5616-3p | 0.35003 | 0.029788 |
| mmu-miR-201-5p | 0.209706 | 0.017856 |
| mmu-miR-3109-5p | 0.253865 | 0.003866 |
| mmu-miR-5121 | 0.446213 | 0.015021 |
| mcmv-miR-m108-1-3p | 0.241742 | 0.002631 |
| mmu-miR-381-5p | 0.307368 | 0.026405 |
| mmu-miR-551b-3p | 0.144723 | 0.024484 |
| mmu-miR-3102-3p | 0.305975 | 0.008883 |

**2. Data Analysis for miRNAs:**

For Analysis of miRNA expression profiling and related data generation are completed with the help of Shanghai Kangcheng Biological Company. And the following are the gene expression values were plotted**: Briefly, 1. Data Analysis for miRNAs:** Low intensity filtering and data normalization. Scanned images were then imported into GenePix Pro 6.0 software (Axon) for grid alignment and data extraction. Replicated miRNAs were averaged and miRNAs that intensities>=30 in all samples were chosen for calculating Median normalization factor. Expressed miRNA data were normalized using the Median normalization and chosen for differentially expressed miRNAs screening. After normalization, the distributions of log2-ratios across every sample are nearly the same; To identify differentially expressed miRNAs with statistical significance. The threshold we used to screen Down regulated miRNAs is Fold Change>=2.0 and P-value <=0.05. Following list only represents part of the whole results.
